# Quantification of the temperature equilibrium time of the cavity in parallel-plate-type ionization chambers by thermal analysis

**DOI:** 10.1093/jrr/rrab073

**Published:** 2021-08-16

**Authors:** Hiraku Fuse, Soma Hirota, Tatsuya Fujisaki, Shinji Abe, Kenji Yasue, Koichi Hanada, Fumihiro Tomita

**Affiliations:** Department of Radiological Sciences, Ibaraki Prefectural University of Health Sciences, 4669-2, Ami-machi, Inashiki-gun, Ibaraki 300-0394, Japan; Department of Radiological Sciences, Ibaraki Prefectural University of Health Sciences, 4669-2, Ami-machi, Inashiki-gun, Ibaraki 300-0394, Japan; Department of Radiological Sciences, Ibaraki Prefectural University of Health Sciences, 4669-2, Ami-machi, Inashiki-gun, Ibaraki 300-0394, Japan; Department of Radiological Sciences, Ibaraki Prefectural University of Health Sciences, 4669-2, Ami-machi, Inashiki-gun, Ibaraki 300-0394, Japan; Graduate School of Health Sciences, Department of Radiological Sciences, Ibaraki Prefectural University of Health Sciences, 4669-2, Ami-machi, Inashiki-gun, Ibaraki 300-0394, Japan; Graduate School of Health Sciences, Department of Radiological Sciences, Ibaraki Prefectural University of Health Sciences, 4669-2, Ami-machi, Inashiki-gun, Ibaraki 300-0394, Japan; Graduate School of Health Sciences, Department of Radiological Sciences, Ibaraki Prefectural University of Health Sciences, 4669-2, Ami-machi, Inashiki-gun, Ibaraki 300-0394, Japan

**Keywords:** temperature-equilibrium time, parallel-plate-type ionization chamber, temperature analysis, temperature distribution, absorbed-dose measurement

## Abstract

Temperature corrections are necessary to account for the varying mass of air in the cavity volume of a vented ionization chamber. The temporal response resulting from temperature changes in a cylindrical and/or Farmer-type ionization chamber, which is the standard dosimeter, has been thoroughly discussed by some researchers. The purpose of this study was to characterise and analyse the dependence of the cavity air temperature of the parallel-plate-type ionization chamber on changes in the ambient temperature. Ionization chambers NACP-02 (IBA Dosimetry, GmbH) and Advanced Markus TN34045 (PTW, Freiburg) were modelled using thermal analysis software to present the temperature equilibrium time and the entire ionization chamber temperature distribution. The temporal response of each ionization chamber was measured for comparing the calculation results of the thermal analysis. The ionization chamber cavities of NACP-02 and TN34045 reached complete equilibrium in 670 and 750 s, respectively. Heat transfer occurred faster at the centre of the front wall of TN34045 than at the outside of the centre except for the edges. Further, the non-uniformity of temperature in the cavity was in the range of 24.2–24.8°C for NACP-02 and 23.7–24.4°C for TN34045 at 200 s after the ionization chamber was installed in the water phantom. The previous proposal to wait for about 15 mins after submerging the chamber in a water phantom before the measurement is demonstrated to be appropriate for parallel-plate-type ionization chambers.

## INTRODUCTION

The dose response due to temperature changes in a cylindrical and/or Farmer-type ionization chamber, which is the standard dosimeter, has been well discussed [1–8]. In some studies, a minute thermometer was placed inside the ionization cavity to measure temperature propagation from the outside (i.e. a water phantom or plastic phantom), and in other studies the temperature change inside the ionization cavity was estimated from the amount of ionization. These studies concluded that the time required for temperature equilibrium between the ionization cavity and the outside is about 120 s on average in a Farmer-type ionization chamber. However, since the ionization cavity has a volume, the temperature measurement at a fixed point does not reflect the temperature of the entire ionization cavity. On the other hand, in the thermal analysis in which the Farmer-type ionization chamber was accurately modelled, the temperature equilibrium time was quantified from the thermal conductivity of each substance constituting the ionization chamber [8]. The study showed that it took 400 s to reach complete temperature equilibrium. This result shows that the waiting time (this is the duration of 15 min before measurement for which the ionization chamber is placed in the water phantom), which is conventionally applied, is sufficient. However, the parallel-plate-type ionization chamber for electron beams is not merely a reference dosimeter; it has a complicated internal structure, hence the dose response due to temperature changes has not been fully discussed. In one study, an old-type NACP parallel-plate ionization chamber was used to show the difference in response to temperature changes based on changes in the electric charge [5]. Although the approximate equilibrium time is shown, various error factors, e.g. changes in water temperature during electric charge measurement and measurement accuracy, are involved and more useful data can be obtained if the measurement system is improved.

This study aimed to reproduce two parallel-plate-type ionization chambers using a simulation model for thermal analysis. The thermal analysis was validated by comparing the temporal response calculated from the electric charge obtained from the electric charge measured under identical conditions as in the thermal analysis. From the results of the thermal analysis, we clarified the temperature equilibrium time of the ionization cavity and the temperature distribution of the entire ionization chamber with respect to external temperature changes.

## MATERIALS AND METHODS

### Modelling the parallel-plate-type ionization chamber for thermal analysis

**Fig. 1:**
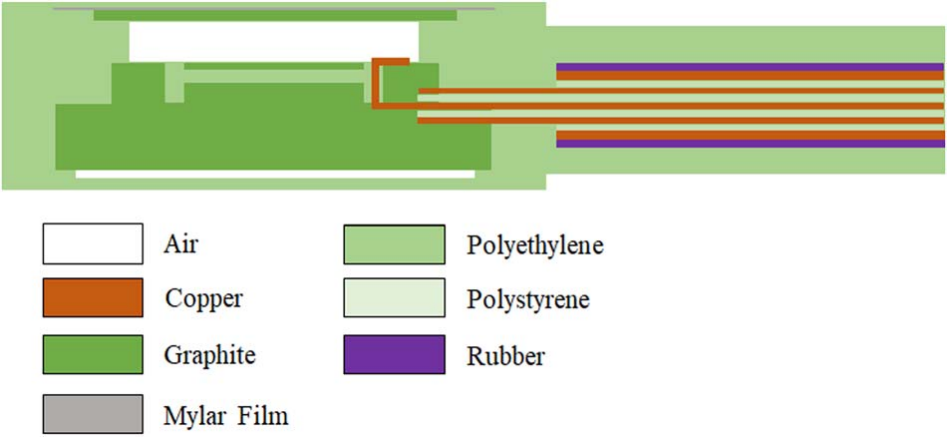
Modelling details of NACP-02. Sensitive volume, cylinder height and guard ring width are 0.16 cm^3^, 2.0 mm and 3.0 mm, respectively. PS and PE stand for polystyrene and polyethylene, respectively.

**Fig. 2:**
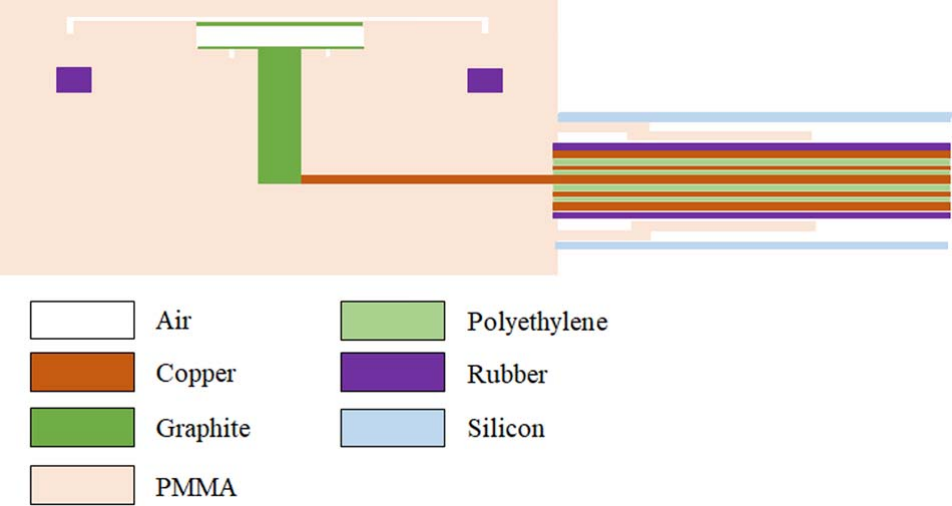
Modelling details of TN34045. Sensitive volume, cylinder height and guard ring width are 0.02 cm^3^, 1.0 mm and 2.0 mm, respectively. PMMA and PE stand for polymethyl-methacrylate and polyethylene, respectively.

Ionization chamber models were created using ThermNet 7.9 (64-bit) (Infolytica Corp., CA, USA) based on the X-ray photographs of NACP-02 (IBA Dosimetry GmbH, Germany) and Advanced Markus TN34045 (PTW GmbH, Freiburg, Germany) and the published structural data [9, 10]. The models are shown in Figs 1 and 2. The materials used for the ionization chamber model are shown in each figure.

### Measurement of temporal response using two ionization chambers

**Fig. 3:**
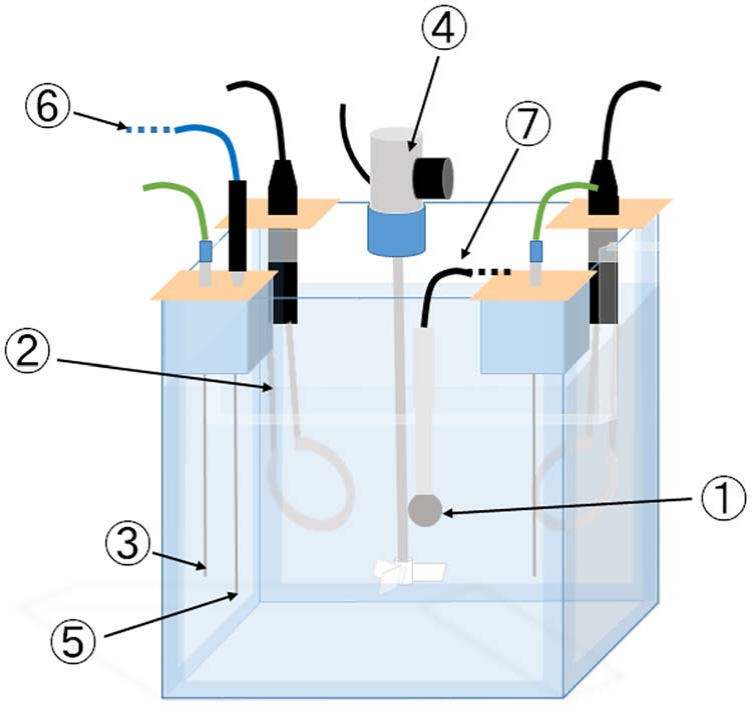
Device layout for measurement. The numbers correspond to the following devices: ① Parallel-plate-type ionization chamber. ② Immersion heater. ③ Digital fine thermostat. ④ Stirrer. ⑤ Digital thermometer. ⑥ Connection to a compact thermologger. ⑦ Connection to an electrometer.

Electric charge measurements were performed using a 12-MeV electron beam with a linac (Elekta Synergy, Elekta AB Stockholm, Sweden) using two ionization chambers based on TRS-398 [11] and the standard dosimetry [12]. The ionization chamber was stored in an incubator set to 20°C the day before the measurement to ensure that the temperature of the ionization chamber was constant at 20°C. A stirrer (AMM-7218, ANEST IWATA Corp., Japan), an immersion heater (A type SWA1505, HAKKO Corp., Japan) and a fine digital thermostat (DG2N-100, HAKKO Corp., Japan) were installed so that the water phantom (T41023, PTW GmbH, Freiburg, Germany) maintained the water temperature at 25°C throughout the experiment. The experimental setup is shown in Fig. 3. At the time of measurement, the two ionization chambers were connected to an electrometer (RAMTEC Duo, TOYO MEDIC, Japan) to read the electric charge. Measurement was performed continuously at 15-s intervals for up to 1000 s. The water temperature and room temperature were acquired by a digital thermometer and a compact thermologger (AM-8000 K, Anritsu Keiki Co., Ltd., Japan).

### Thermal analysis

In the thermal analysis simulation of the parallel-plate-type ionization chamber model, the thermophysical properties of each substance at 20–25°C were referred to from a handbook of their thermophysical characteristics so that the effects of heating and cooling could be reproduced based on the surrounding medium [13]. The thermal analysis was performed with in-house software based on ThermNet 7 (Mentor Graphics Corporation, Wilsonville, OR, USA). Thermal conductivity, material composition and density were entered into this software. In addition, the heat-transfer rate between substances was calculated from the following equation and input to the software:

(1) \begin{equation*} {N}_{\mathrm{u}}=\frac{h{L}_{\mathrm{ch}}}{k} \end{equation*}

where ${N}_{\mathrm{u}}$ denoted the Nusselt number, *k* [W/mK] denoted a heat-conductivity coefficient, *h* [W/m^2^K] denoted a heat-transfer coefficient and *L*_ch_ [mm] denoted length of subject. Table 1 lists the materials and the thermal conductivity values for the parallel-plate-type ionization chambers shown in Figs 1 and 2. The chamber was installed in a water phantom, and the temperature distribution inside the chamber at 20–25°C was analysed. For NACP-02 and TN34045, the temperatures at the centre of the ionization cavity and at a distance of 4 mm from the centre to the exterior of the cavity were calculated. The temperature equilibrium time was determined from these temperatures. The commissioning was confirmed in a centre with simulated temperature and measured average temporal response to ensure the validity of the analysis. The analysis error was set so that the time resolution was 0.01 s, and the Newton tolerance was 0.01 or less.

**Table 1:** Materials and thermal conductivity used in the two parallel-plate-type ionization chambers

Material	Thermal conductivity
	(W/m K) at 22°C^13^
PMMA	0.35
AIR	0.026
Graphite	100
Mylar Film	0.14
PS	0.13
Copper	398
PE	0.46
Rubber	0.13
Silicon	0.5
Aluminium	236

## RESULTS

**Fig. 4:**
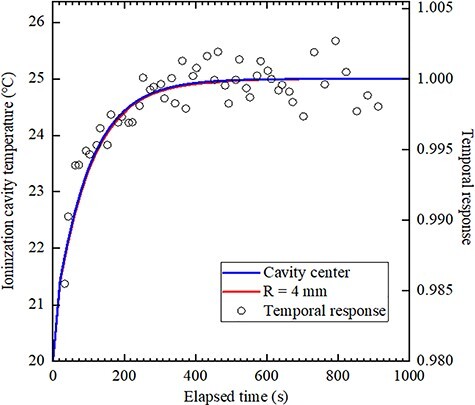
Temporal response and temperature change of the ionized cavity of NACP-02 as obtained from the thermal analysis. Blue and red lines indicate the centre of the cavity and a distance of 4 mm from the centre of the cavity, respectively.

**Fig. 5:**
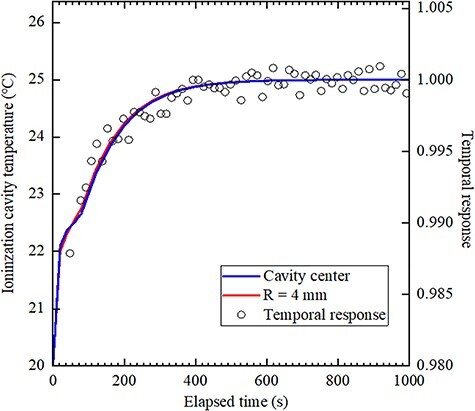
Temporal response and temperature change of the ionized cavity of TN34045 as obtained from the thermal analysis. Blue and red lines indicate the centre of the cavity and a distance of 4 mm from the centre of cavity, respectively.

Figs 4 and 5 show the temporal response and the temperature change in the ionized cavity as obtained from the thermal analysis for each ionization chamber. This result shows that the temperature difference between the ionization cavity and water is 5°C. Here, the temporal response was measured with an accuracy of 0.5%, and the temperature of each location was calculated so that the error was 0.6%. The first vertical axis in the Figs 4 and 5 shows the relationship of the temperature of the ionized cavity obtained from the thermal analysis, and the second vertical axis in Figs 4 and 5 shows the relationship of the temporal response obtained by measurement. Regarding the temporal response, the average value after 700 s was set to 1. For NACP-02, there was no difference between the temperature change at the centre of the ionization cavity and at a distance of 4 mm from the centre. For TN34045, a comparison of temperature change at the centre of the ionization cavity and at a distance of 4 mm from the centre showed a difference of up to 1.8% (5 s) in 80–300 s.

NACP-02 reached complete equilibrium in about 670 s. The time constant calculated from Fig. 4 is 86 s. Assuming that the equilibrium time is five times the time constant, it is 430 s. TN34045 reached complete equilibrium in about 750 s. The time constant calculated from Fig. 5 is 108 s, and the equilibrium time calculated as five times the time constant is 540 s. In the results obtained by the thermal analysis, the inflection points that could not be obtained by electric charge measurement were observed within 30–100 s in the case of TN34045.

**Fig. 6:**
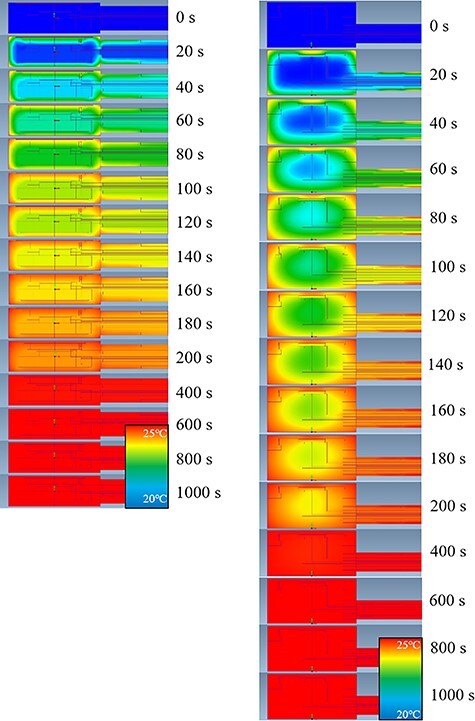
Temperature distribution of the entire ionization chambers of NACP-02 (left) and TN-34045 (right) as obtained by thermal analysis. The interval is 20 s, from 0 to 200 s for large changes in temperature.

The temperature distribution of the entire ionization chamber of NACP-02 and TN34045 is shown in Fig. 6. NACP-02 warmed from the front wall, and it took time for the chamber centre that is composed of graphite to warm up on the rear side. TN34045 also warmed from the front wall. In addition, the temperature of the marginal part of the ionization cavity rose faster than that of the central part.

## DISCUSSION

The complete temperature equilibrium time of the two parallel-plate-type ionization chambers in this study was about 670 s for NACP-02 and about 750 s for TN34045. The temperature equilibrium time was calculated with the time constant. The time constant was 86 s and 108 s for NACP-02 and TN34045, respectively. When considering the equilibrium time as the fifth time constant, the equilibrium time is 420 s and 540 s for NACP-02 and TN34045, respectively. In the study of an old-model NACP by Almasri *et al.* [5], we can calculate with approximately 300 s at complete equilibrium time from the fifth time constant of their measurement data. Although they also studied the temperature change via measurement, the difference between their results and the result of this study is attributed to the unevenness of measurements due to temperature control of the water phantom. However, the measured values ​​of NACP-02 also varied at around 25°C in this study. The reason for this was the difference between NACP-02 and TN34045 is the thickness of the front wall. It is considered that NACP-02, which has a thin front wall, was more affected by slight changes in water temperature than TN34045. Considering the temperature equilibrium of the Farmer-type ionization chamber, measurements are performed after waiting for around 15 min after setting up the ionization chamber in the water phantom and/or the plastic phantom in many facilities; this appears to be a suitable waiting time for a parallel-plate-type ionization chamber.

Two steps up were shown in the temperature rise in the ionization cavity in the two ionization chambers as shown in Figs 4 and 5. Although this result has not been discussed so far, the same explanation as that provided for the temperature analysis of the Farmer-type ionization chamber may be considered [8]. First, the ionization cavity is affected by the heat from the front wall; it is inferred that the subsequent heat transfer from a back wall or a lateral wall also influences the temperature change of the ionization cavity. This phenomenon is clearly observed in all parallel-plate-type ionization chambers when large differences exist between the front and back walls and lateral heat transfer occurs. The front wall in NACP-02 is thinner than that in TN34045, and the ionization cavity warms up earlier in the former. In addition, NACP-02 has a smaller temperature equilibrium time because of its smaller volume, including the back wall. Besides, in NACP-02, the rear wall is made of polystyrene and the interior, of graphite. In contrast, the surface as well as bulk of TN34045 is composed of PMMA. Because graphite has a higher thermal conductivity than PMMA, the ionization cavity warms faster in NACP-02.

**Fig. 7:**
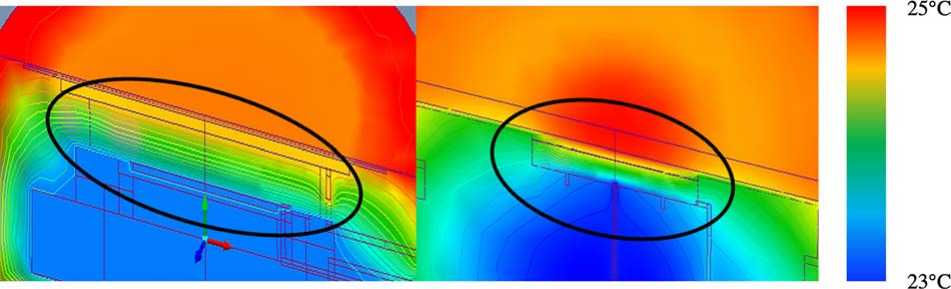
Temperature distribution of the neighbourhood of the cavity. Circles indicate the modelled cavity region.

The temperature distribution was analysed for the two ionization chambers. Due to the volume of the ionization cavity, temperature measurements at fixed points as previous study do not reflect the temperature of the entire ionization cavity. By solving this and showing the overall temperature distribution, the difference in heat transfer between the ionization chambers was clarified. The graphite in NACP-02 and PMMA in TN34045 have significantly different thermal conductivities. Thermal analysis clarified that the ionization cavities of the two chambers differed in the time taken to attain the same temperature by about 40 s. Detailed analysis of the temperature distribution inside the cavity showed that the temperature inside the cavity was in the range 24.2–24.8°C for NACP-02 and 23.7–24.4°C for TN34045 at 200 s after the ionization chamber was installed in the water phantom (Fig. 7). A temperature difference of 0.6–0.7°C causes uncertainty of approximately 0.25% in the measurement. Hence, it is necessary to measure the charge after the interior of the ionization cavity has attained temperature equilibrium with the outside. Further, the two chambers differed in terms of the heat transfer from the front wall. The front wall of the ionization cavity was uniformly warmed in NACP, whereas the heat transfer was higher at the centre of the front wall of the ionization cavity than at the edge of in TN34045 (see Fig. 7). This difference arises from the detachability of the front wall of TN34045, which is attributed to the presence of an air layer between the cavity and front wall [10]; this is not the case in NACP-02 [9]. The diamorphism the temperature curve in the thermal analysis is clarified from the temperature distribution.

## CONCLUSION

Two parallel-plate-type ionization chambers were modelled for thermal analysis, and the temperature equilibrium time and temperature distribution of the ionization chamber with respect to external temperature changes were examined. The ionization cavities of NACP-02 and TN34045 attained complete equilibrium time in 670 and 750 s, respectively. However, during temperature rise, the temperature distribution in the ionization cavity is not uniform at times, and it is necessary to secure a sufficient waiting time before starting the measurement. The previous proposal to wait for about 15 min after submerging the chamber in a water phantom before the measurement is appropriate for parallel-plate-type ionization chambers.
